# Supplementary material for: Structural characterization of DNA-binding domain of essential mammalian protein TTF 1
Source: Biosci Rep. 2024 Aug 28;44(8):BSR20240800. doi: 10.1042/BSR20240800 (PMC11358750; doi:10.1042/BSR20240800)
Supplement: Supplementary Figures S1-S5 and Supplementary Box S1 [file BSR-2024-0800_supp.pdf]

Supplementary information

Box 1: Amino acid sequence of Myb domain of TTF1

Q62187 Uniprot ID of mouse TTF1

Myb Domain: 550 to 732 amino acids

Sequence:
TLITNLKRKHAFRLHIGKGIARPWKLVYYRAKKIFDVNNYKGRY
NEEDTKKLKAYHSLHGNDWKKIGAMVARSSLSVALKFSQIGGTR
NQGAWSKAETQRLIKAVEDVILKKMSPQELRELDSKLQEDPEGR
LSIVREKLYKGISVEARVETRNWMQCKSKWTEILTKRMTHGGFV
YRGV

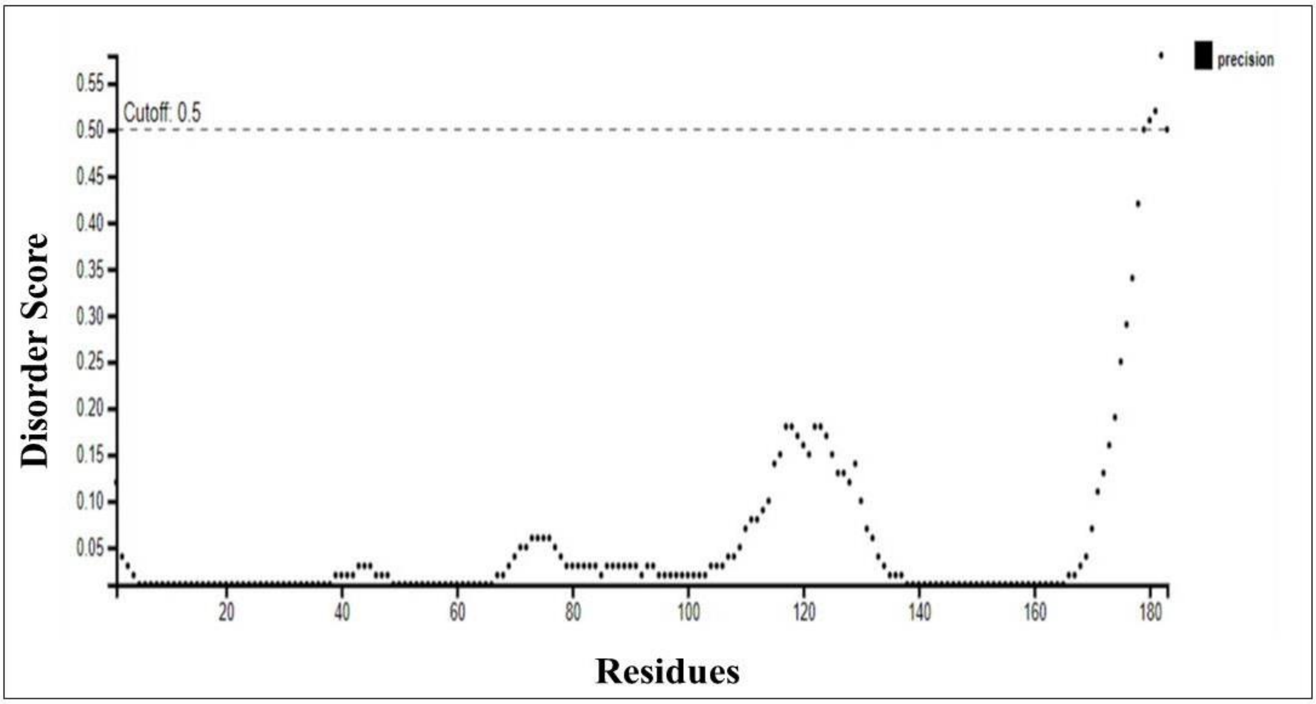

Figure S1: Graphical representation of disordered Myb domain regions in mouse TTF1 protein. Residues with disorder score ≥ 0.5 were considered to be disordered.

```
TTF1
TLITNLKRKHAFRLHIGKGIARPWKLVYYRAKKIFDVNNYKGRYNEEDTKKLKAYHS
LHG      60
Reb1     ---------------------------------PF---EDRCVWSKEEDEELRKNVVEHG         24
                                *   : :  :..:*: ::*:     **

TTF1                                               NDWKKIGAMVARSSLSVALKF---
SQIGGTRNQGAWSKAETQRLIKAVEDVILKKMSPQE          117
Reb1
KCWTKIGRKMARMPNDCRDRWRDVVRFGDKLKRNAWSLEEETQLLQIVAEL---------  75
          : *.***  :**    .   ::    ::*.. ::.***  *  :*:: * ::

TTF1
LRELDSKLQEDPEGRLSIVREKLYKGISWVEVEARVETRNWMQCKSKWTEILTKRMT
HGG      177
Reb1     -----------------RNREDLSSDINWTLVAQMLGTRTRLQCRYKFQQLTKAAS----      114
                       **.* ..*.*. *   : **. :**: *: :: .

TTF1     FVYRGV            183
Reb1     ------       114
```

Figure S2: Sequence alignment of the Myb domain of mouse TTF1 and Reb1 protein

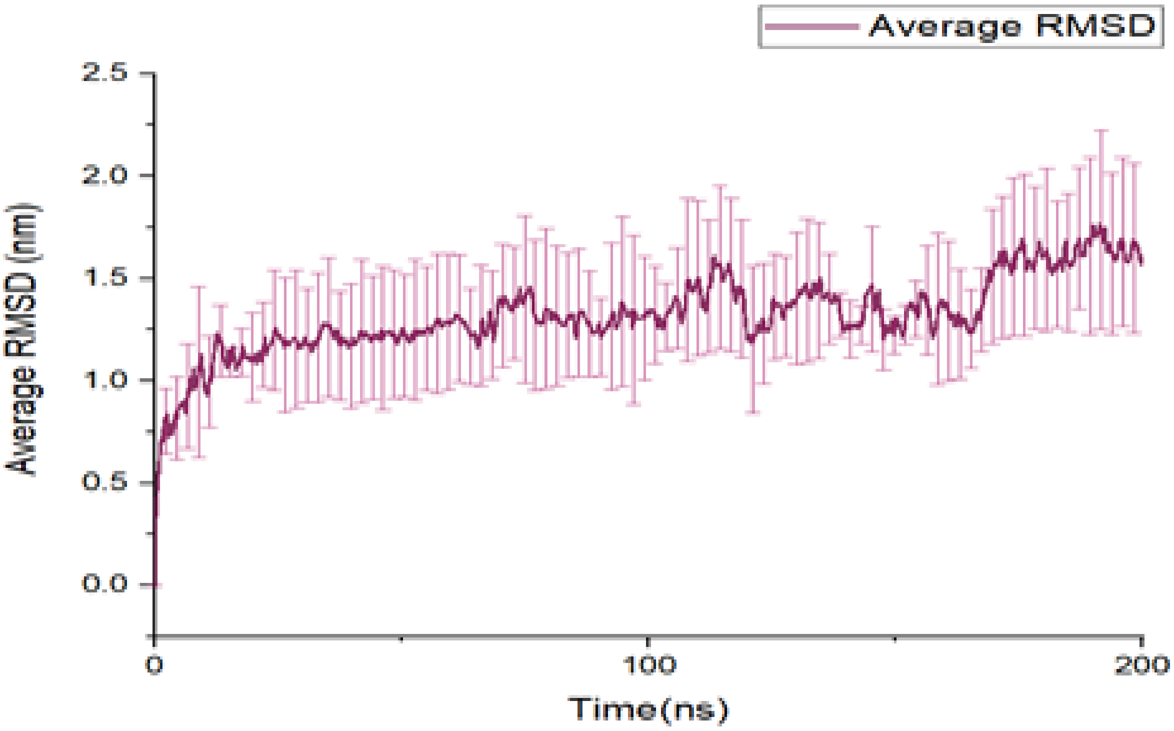

Figure S3: Average of RMSD value for triplicate run.

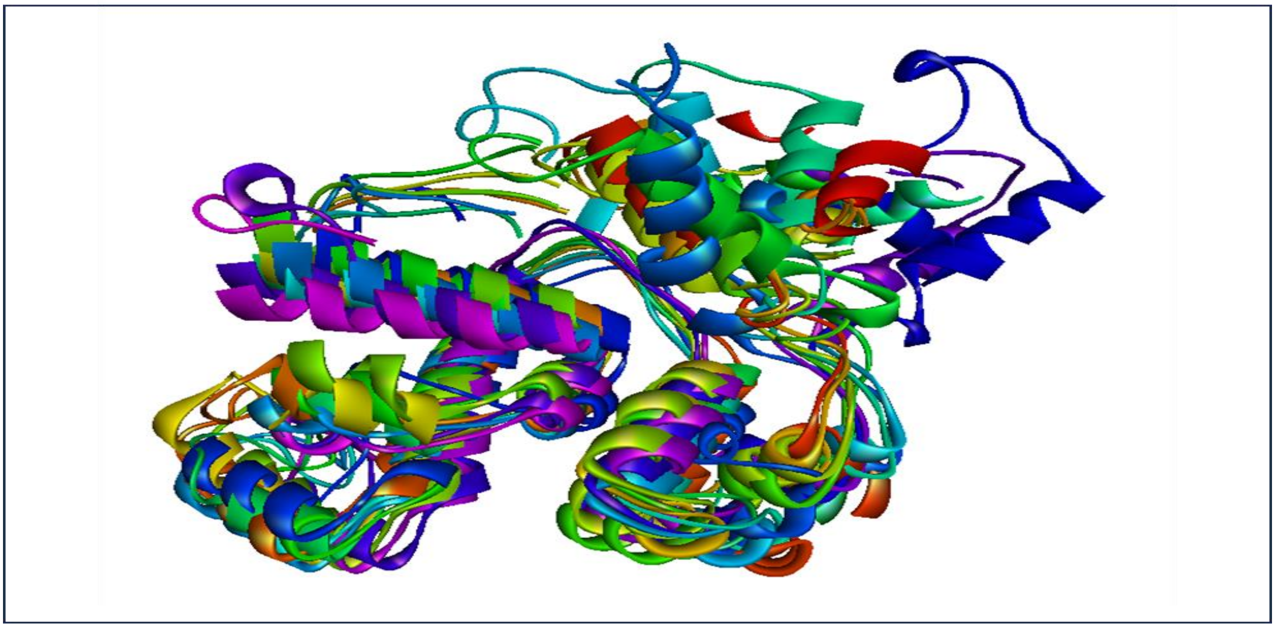

Figure S4: The overlay of protein structure after every 20 ns. Frame for protein after every 20 ns were extracted and align using PyMOL

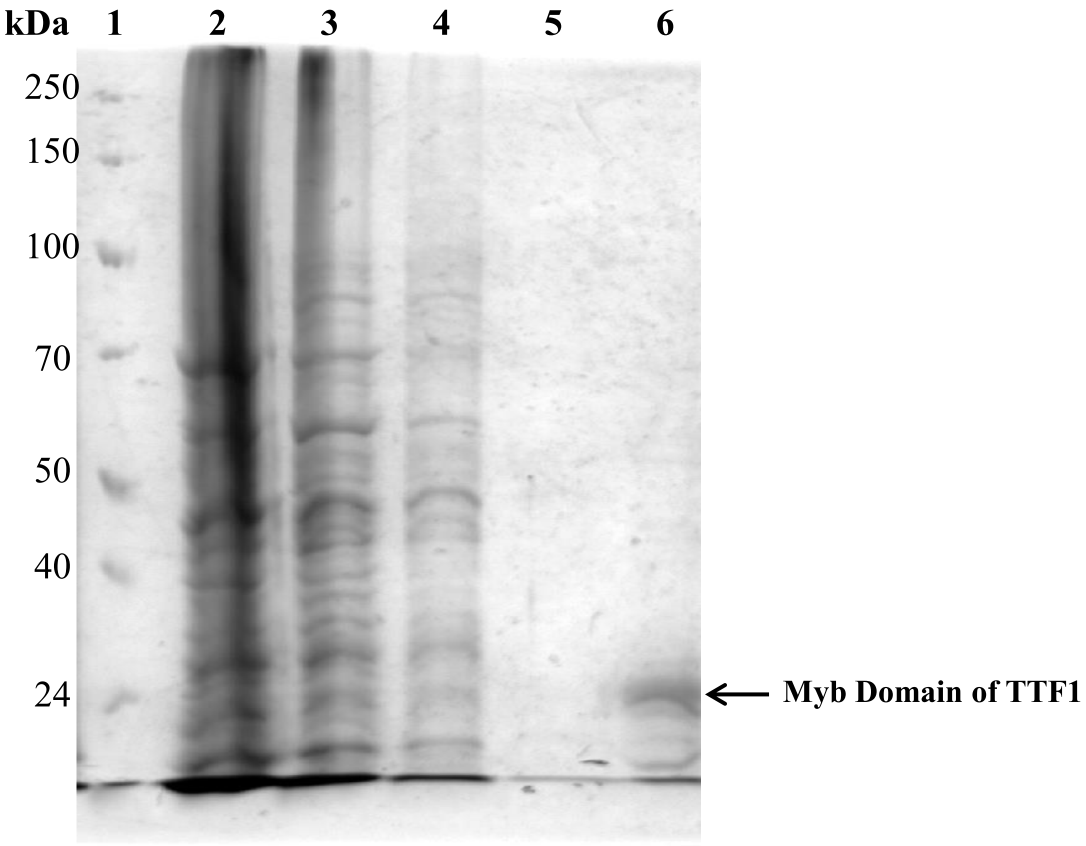

Figure S5: SDS-PAGE profile of the affinity purified protein. Lane 1 protein marker, Lane 2 Cell lysate, Lane 3 Flow through, Lane 4 &5 Wash 1 and Wash 2, Lane 6 Elution 1.
